# Supplementary material for: Fractionalized excitations in the partially magnetized spin liquid candidate YbMgGaO4
Source: Nat Commun. 2018 Oct 8;9:4138. doi: 10.1038/s41467-018-06588-1 (PMC6175835; doi:10.1038/s41467-018-06588-1)
Supplement: Supplementary file 1 — Supplementary Information [file 41467_2018_6588_MOESM1_ESM.pdf]

Supplementary Information for

Fractionalized excitations in the partially magnetized spin liquid candidate $YbMgGaO_4$

Shen *et al.*

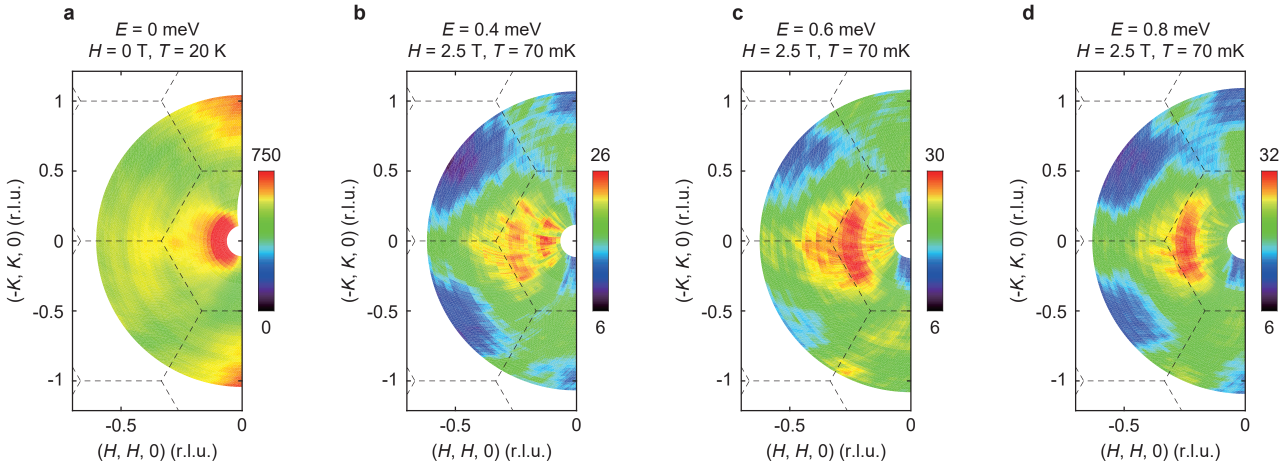

Supplementary Figure 1: **Correction of neutron beam self-attenuation. a,** Elastic incoherent scattering image at 20 K and 0 T. **b-d,** Raw constant-energy images at the indicated energies. Dashed lines indicate the Brillouin zone boundaries. The colour bar indicates scattering intensity in arbitrary unit in linear scale.

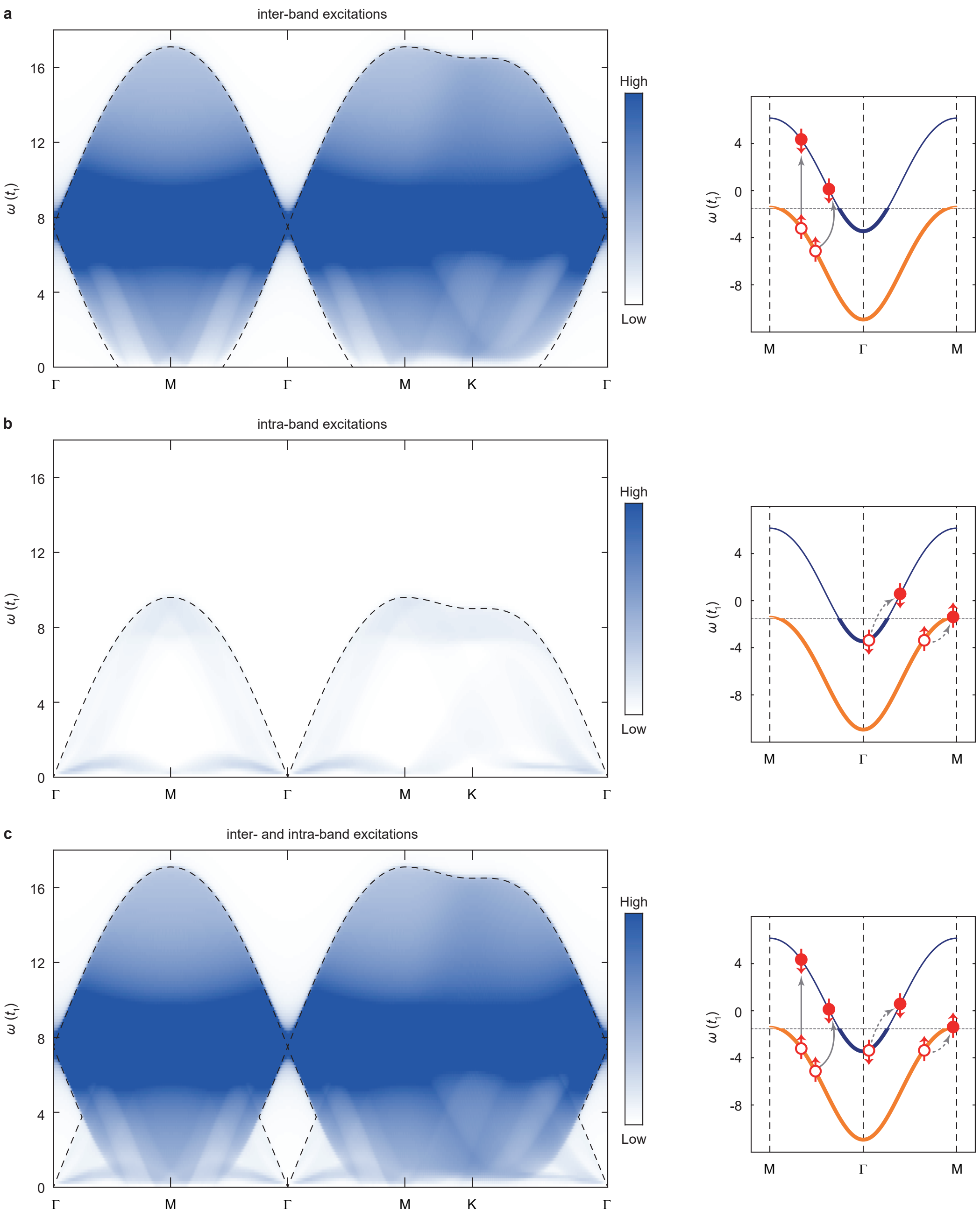

Supplementary Figure 2: **Calculation of the spinon band structure and its excitations in a weak magnetic field. a,** The dynamic spin structure factor $\mathcal{S}_{+-}(\boldsymbol{p}, E)$ defined in Eq. 3 (left) in the main text and illustration of the vertical and tilted inter-band particle-hole excitations (right). These are the dominant events that are responsible for the spectral peak at (Γ, Δ) and the upper and lower excitation edges that cross at the peak. **b,** The dynamic spin structure factor $\mathcal{S}_{zz}(\boldsymbol{p}, E)$ defined in Eq. 4 (left) in the main text and illustration of the intra-band particle-hole excitations (right). These are analogous to the particle-hole excitations in the zero-field case, and also gives rise to an upper excitation edge at Γ. **c,** The combined dynamic spin structure factur defined in Eq. 2 in the main that is proportional to the observed neutron scattering density. In all the figures we chose t_2/t_1 to be 0.2 and Zeeman splitting gap $\Delta = 7.5t_1$ where t_1 and t_2 indicate the nearest and next-nearest neighbour spinon hoppings. The colour bar indicates scattering intensity in arbitrary unit in linear scale.
